# Application of a Novel Microtitre Plate-Based Assay for the Discovery of New Inhibitors of DNA Gyrase and DNA Topoisomerase VI

**DOI:** 10.1371/journal.pone.0058010

**Published:** 2013-02-26

**Authors:** James A. Taylor, Lesley A. Mitchenall, Martin Rejzek, Robert A. Field, Anthony Maxwell

**Affiliations:** Department of Biological Chemistry, John Innes Centre, Colney Lane, Norwich, United Kingdom; University of Minnesota, United States of America

## Abstract

DNA topoisomerases are highly exploited targets for antimicrobial drugs. The spread of antibiotic resistance represents a significant threat to public health and necessitates the discovery of inhibitors that target topoisomerases in novel ways. However, the traditional assays for topoisomerase activity are not suitable for the high-throughput approaches necessary for drug discovery. In this study we validate a novel assay for screening topoisomerase inhibitors. A library of 960 compounds was screened against *Escherichia coli* DNA gyrase and archaeal *Methanosarcina mazei* DNA topoisomerase VI. Several novel inhibitors were identified for both enzymes, and subsequently characterised *in vitro* and *in vivo*. Inhibitors from the *M. mazei* topoisomerase VI screen were tested for their ability to inhibit Arabidopsis topoisomerase VI in planta. The data from this work present new options for antibiotic drug discovery and provide insight into the mechanism of topoisomerase VI.

## Introduction

The emergence of pathogenic bacterial strains resistant to currently available antimicrobial agents is a universal problem of mounting importance [1]–[3]. Resistance mechanisms have been reported for all known classes of antibiotics with some strains exhibiting multiple resistance phenotypes, which is a consequence of natural selection and human mismanagement [4]. The danger that these strains pose is demonstrated by the increased mortality and morbidity rates for infected patients when compared to those infected with susceptible strains [5], [6]. Unfortunately this increase in resistance has not been met with an increase in the development of new antibiotics, with the total number of new drugs being brought to market actually decreasing [7]. Clearly there is an urgent need for the development of new antibiotics and management strategies.

Extensive attempts to validate new target enzymes for antimicrobials have met with little success [8], with the majority of successful drugs inhibiting a handful of cellular processes. One of the most successfully exploited drug targets is the DNA topoisomerase (topo) class of enzymes [9]–[12]. DNA topoisomerases are essential and ubiquitous enzymes responsible for controlling the topological state of DNA [13]. This is accomplished by the reaction of an active-site tyrosine with the phosphate backbone of the DNA to generate a covalent intermediate (the so-called ‘cleavage complex’), followed by either strand passage of another segment of DNA or free rotation of the broken strand [14]–[17]. DNA topoisomerases are classified as either type I or type II based on whether they cleave one or both strands of the DNA [18], and further subdivided into IA, IB, IC, IIA or IIB based on structural and mechanistic differences [19]. The essential nature of these enzymes and the vulnerability of the cleavage complex, which, if stabilised, rapidly results in cell death, make them ideal drug targets.

The type IIA topoisomerases have been the most exploited class, acting as targets for many anticancer and antibacterial drugs. DNA gyrase is a type IIA topoisomerase of particular importance due to it being a target for numerous antibacterial drugs and its distinct mechanism. All type IIA topoisomerases are capable of removing supercoils from DNA (relaxation) in an ATP-dependent manner [20]; gyrase introduces negative supercoils into DNA in the presence of ATP, but relaxes DNA when ATP is absent [21]. Whereas eukaryotic type IIA topoisomerases are dimeric in nature, gyrase forms a heterotetramer of two GyrB subunits, which contain the ATPase domains, and two GyrA subunits, which contain the active-site tyrosines [22]. During the reaction cycle, the segment of DNA to be cleaved (the ‘gate’ or ‘G’ segment) binds to the DNA-binding saddle in GyrA. ATP binding causes the GyrB subunits to dimerise and capture a second segment of DNA (the ‘transported’ or ‘T’ segment) [23]. The G segment is then cleaved and the break pried open by conformational changes, allowing the T segment to pass through. The G segment can then be religated. The differences in mechanism and structure between gyrase and eukaryotic topoisomerases, as well as its low homology to human type IIA topoisomerases, have allowed the development of bactericidal drugs that target bacterial topoisomerases with a high degree of specificity.

The mechanism of action for DNA gyrase inhibitors is highly varied, with different chemical families inhibiting different steps in the reaction cycle [12]. The most successful class of gyrase inhibitors is the “billion-dollar” quinolone family of drugs. Quinolones have the potent ability to stabilise the cleavage complex of DNA gyrase, resulting in double-strand breaks and cell death [24], [25]. The exact mechanism by which this occurs remains unclear, but several crystal structures of quinolones bound to gyrase or its sister enzyme topo IV have been published [26]–[29]. These structures suggest that quinolones bind in pockets near the active-site tyrosines while simultaneously intercalating with the cleaved DNA, presumably distorting it in such a way as to prevent religation. In contrast, the aminocoumarin class of inhibitors target the ATPase activity of the enzyme in a competitive manner, binding in a pocket that overlaps with the ATP-binding site and sterically hindering nucleotide binding [30]. Unfortunately these compounds possess unfavourable pharmacokinetics and produce too many side effects to be effective clinical antimicrobials [31], [32]. The simocyclinone class of inhibitors prevents the binding of the enzyme to DNA by a novel “double-warhead” mechanism [33], [34]. These compounds consist of an aminocoumarin group linked to a polyketide group by a long linker, both groups bind to two separate sites in the DNA-binding saddle of GyrA. A crystal structure of the drug bound to GyrA shows two GyrA dimers being cross-linked by four drug molecules, occluding the DNA-binding saddle [34]. However an alternative model, supported by mass spectrometry data, has been proposed in which one molecule of SD8 bridges the two sites on the same GyrA subunit, hindering DNA binding [35].

The type IIB family of topoisomerases has similarities to the type IIA family, but has significant structural and mechanistic differences. The family currently consists of a single enzyme: topo VI. Similar to the bacterial type IIA enzymes topo VI is an A_2_B_2_ heterotetramer, with the ATPase sites located on the B subunits and the active-site tyrosines located on the A subunit [36], [37]. The A subunits of topo VI lack some of the extensive protein-protein interactions of the type IIA enzymes. In addition, whereas the type IIAs are capable of cleaving DNA in the absence of ATP, ATP hydrolysis and DNA cleavage have been shown to be tightly coupled during the reaction cycle of topo VI from the thermophilic archaea *Sulfolobus shibatae* [38]. It has been suggested that these two features evolved in parallel, with the reduction in protein-protein interactions necessitating tighter coupling between strand cleavage and ATP binding in order to prevent accidental double-strand lesion formation [23].

In contrast to the type IIA topoisomerases, the type IIB family has received relatively little attention, being regarded for many years an “archaeal curiosity”. However the discovery of topo VI homologues in plants [39]–[41] and the malaria parasite *Plasmodium falciparum* [42] opens the possibility for this enzyme being used as a target for herbicides or anti-malarial agents. In plants, topo VI has been shown to play a key role in the process of endoreduplication [39], [40], in which the plant cell replicates its genome several times without dividing, accompanied by cell expansion. Knock-out mutants of topo VI in *Arabidopsis thaliana* have an extreme dwarf phenotype, yellowish leaves, reduced trichome (“leaf hair”) size, reduced chromosome counts (ploidy) and reduced root hair size and frequency [39], [40]. These plants die after 4–5 weeks of growth, which indicates the essential nature of topo VI in plants.

Neither plant nor malarial topo VI have been successfully purified, but extensive work has been carried out on the *Sulfolobus shibatae* orthologue of the enzyme. Unlike the type IIA topoisomerases, high-resolution crystal structures of the entire A_2_B_2_ complex have been solved for *S. shibatae* topo VI and the topo VI from the methanogenic archeon *Methanosarcina mazei* [36], [37]. A few inhibitors have been identified for the *S. shibatae* enzyme, including the Hsp90 inhibitor radicicol, which has been shown to inhibit the ATPase activity of the enzyme [43]. In addition, several inhibitors of eukaryotic topo II have been shown to inhibit *S. shibatae* topo VI, although their mechanism of action has not been determined [44]. Novel inhibitors targeting topo VI will be invaluable as probes of the enzyme's mechanism and may well provide the basis for novel chemotherapeutics.

Despite their great potential as drug targets, high-throughput screening for inhibitors of topoisomerases has been limited by the traditional assays for topoisomerase activity, which are poorly suited to rapidly processing large numbers of reactions in a quantitative fashion. We have previously described a novel microtitre plate-based assay that allows the processing of a large number of reactions simultaneously and has the potential to be automated [45]–[47]. This assay is based on the observation that supercoiled plasmids form intermolecular DNA triplexes more readily than relaxed plasmids. A triplex-forming oligonucleotide is immobilised on a microtitre plate surface and used to capture supercoiled plasmids from solution, which can be subsequently stained with a fluorescent dye. This in turn allows for the supercoiling or relaxation activity of topoisomerases to be monitored.

Although this assay has been validated in a low-throughput context, its efficacy in a high-throughput context has yet to be reported. We have conducted a proof-of-principle screen of a library of 960 compounds, consisting of 80% FDA-approved drugs and 20% natural products, against DNA gyrase from *E. coli* and *M. mazei* topo VI. It was expected that by screening against compounds already shown to interact with biological molecules the screen would achieve a higher hit rate than with a random library [48]–[50]. *M. mazei* topo VI was selected over the *S. shibatae* orthologue since the thermophilic nature of the *S. shibatae* enzyme makes it a potentially less relevant model for eukaryotic topo VI enzymes. Furthermore, few inhibitors have been so far discovered for this enzyme. The mechanisms of action of the hits from both screens were explored *in vitro*, and their antibiotic properties assessed in cell-based assays against Gram-positive and Gram-negative bacteria, for the DNA gyrase hits, or against *Arabidopsis thaliana*, for the topo VI hits.

## Results

### Screening a chemical library against *E. coli* gyrase and *M. mazei* topoisomerase VI

**Figure 1:**
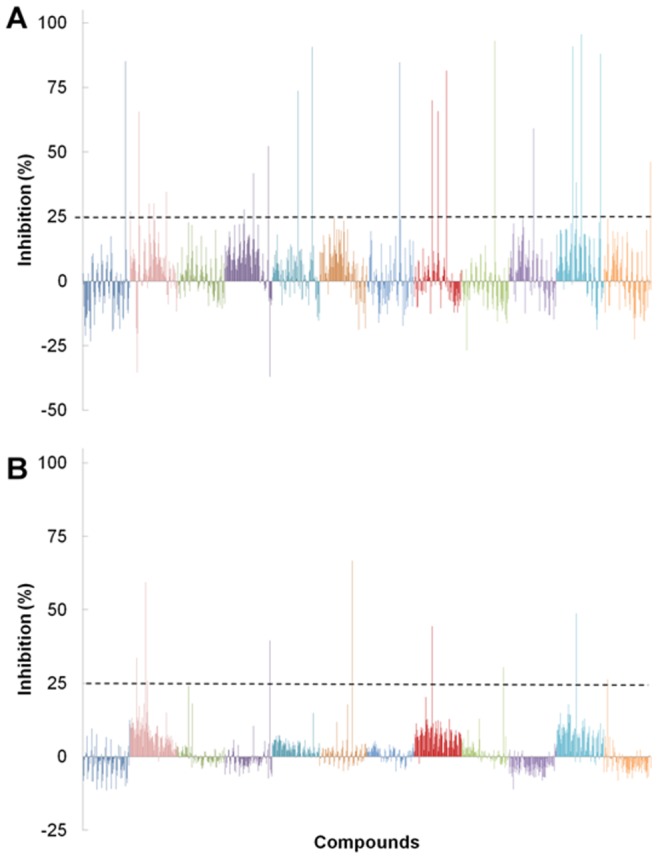
Screening the Microsource GenPlus chemical library against *E. coli* DNA gyrase and *M. mazei* DNA topoisomerase VI. A. Results from the DNA gyrase screen. B. Results from the topoisomerase VI screen. Each bar represents the average percentage inhibition for a compound screened in duplicate. Compound bars have been coloured to indicate 96-well plate groupings. The arbitrary hit threshold of 25% inhibition is demonstrated by the dotted lines.

Both screens were conducted in duplicate over three days at a compound concentration of 25 µM (Figure 1). A hit threshold of 25% inhibition was set (based upon our previous unpublished data), and any compounds exceeding this limit were validated using agarose gel assays (DNA supercoiling or relaxation). The quality of the screening data was determined by calculating the mean fluorescent signals and standard deviations for the 192 negative (DNA alone) and 192 positive (DNA plus enzyme with no drug) controls in each screen. The signal-to-background ratio was calculated to be 5 for the gyrase screen and 4 for the topo VI screen, while the signal-to-noise ratio was 10 for the gyrase screen and 15 for the topo VI screen. The overall quality of the data for both screens was good, with both having an average Z' factor of above 0.5 and no obvious patterns in the data [51]. The average of the Z' factors for the twelve plates was calculated to be 0.64 for the gyrase screen and 0.69 for the topo VI screen, indicating that there was a good degree of separation between the positive and negative controls and implying a good overall quality of the data. The distribution of the Z' factor for each plate around the mean was close, with no single plate giving a value below 0.4.

**Figure 2:**
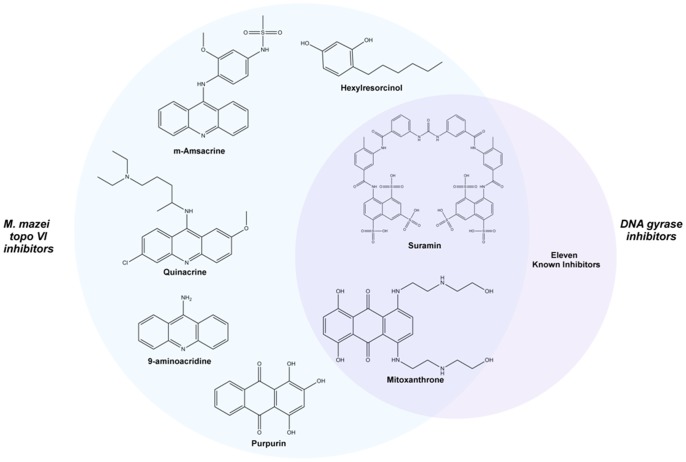
Structures of screen hits. The *M. mazei* topo VI inhibitors are circled in blue on the left and the DNA gyrase inhibitors are circled in purple on the right. The 11 already well characterised inhibitors of DNA gyrase identified by the screen are not shown.

In the gyrase inhibitor screen, 22 compounds scored over the hit threshold (Table 1 and Figure 2). The majority of these were already characterised as DNA gyrase inhibitors including a number of fluoroquinolones, novobiocin and acriflavinium [12], [52], which were not studied further. Out of the remaining hits, mitoxantrone and suramin displayed inhibition while the other 9 compounds tested did not significantly affect DNA gyrase activity in the gel-based supercoiling assay. This gave 13 validated hits, resulting in a hit rate of 1.35% and a novel hit rate of 0.21%. Four known gyrase inhibitors were missed by the screen: nalidixic acid, cinoxacin, oxolinic acid and enoxacin. These false negatives were likely to be due to low inhibitor potency.

**Table 1:** Summary of Screen Hits.

Compound	Target	Approx. IC_50_ (µM)	Mechanism
Novobiocin	DNA gyrase	N.D.*	Aminocoumarin ATPase inhibitor
Acriflavinium	DNA gyrase	N.D.*	ATPase inhibitor
Ciprofloxacin	DNA gyrase	N.D.*	Fluoroquinolone cleavage-complex stabiliser
Gatifloxacin	DNA gyrase	N.D.*	Fluoroquinolone cleavage-complex stabiliser
Levofloxacin	DNA gyrase	N.D.*	Fluoroquinolone cleavage-complex stabiliser
Lomefloxacin	DNA gyrase	N.D.*	Fluoroquinolone cleavage-complex stabiliser
Moxifloxacin	DNA gyrase	N.D.*	Fluoroquinolone cleavage-complex stabiliser
Norfloxacin	DNA gyrase	N.D.*	Fluoroquinolone cleavage-complex stabiliser
Ofloxacin	DNA gyrase	N.D.*	Fluoroquinolone cleavage-complex stabiliser
Perfloxacine Mesylate	DNA gyrase	N.D.*	Fluoroquinolone cleavage-complex stabiliser
Surafloxacin	DNA gyrase	N.D.*	Fluoroquinolone cleavage-complex stabiliser
Mitoxantrone	DNA gyrase/Topo VI	12/2	Cleavage-complex stabiliser/Prevents cleavage
Suramin	DNA gyrase/Topo VI	80/30	Blocks DNA binding
Purpurin	Topo VI	40	Blocks DNA binding
9-Aminoacridne	Topo VI	6	Prevents DNA cleavage
Quinacrine	Topo VI	8	Prevents DNA cleavage
m-Amsacrine	Topo VI	30	Prevents DNA cleavage
Hexylresorcinol	Topo VI	30	Unknown

* Not determined in this study.

For the *M. mazei* topo VI screen, 9 compounds that exceeded the hit threshold were selected for further study (Figure 1B). Out of these only m-amsacrine had been previously reported as an inhibitor of topo VI [53], and only against the *S. shibatae* enzyme. Six of these 9 were validated as hits in the gel-based relaxation assay: m-amsacrine, suramin, hexylresorcinol, 9-aminoacridine, purpurin and quinacrine (Figure 2). This gave a hit rate of 0.63%. Of these compounds m-amsacrine, suramin and quinacrine have been previously shown to inhibit type IIA topoisomerases [54]–[56], whilst purpurin and 9-aminoacridine are structurally related to known topo II inhibitors (mitoxantrone and m-amsacrine respectively). Mitoxantrone was subsequently found to inhibit *M. mazei* topo VI, this was missed during the initial screen due to its disruptive effects on DNA triplex formation; this illustrates a potential limitation of this assay. No false negatives were identified but since there are few known inhibitors described for topo VI, this analysis was less informative than with the DNA gyrase screen. Apart from m-amsacrine, none of the other previously described inhibitors [44], [57] were present in the library.

### Mitoxantrone and suramin are novel inhibitors of *E. coli* gyrase

**Figure 3:**
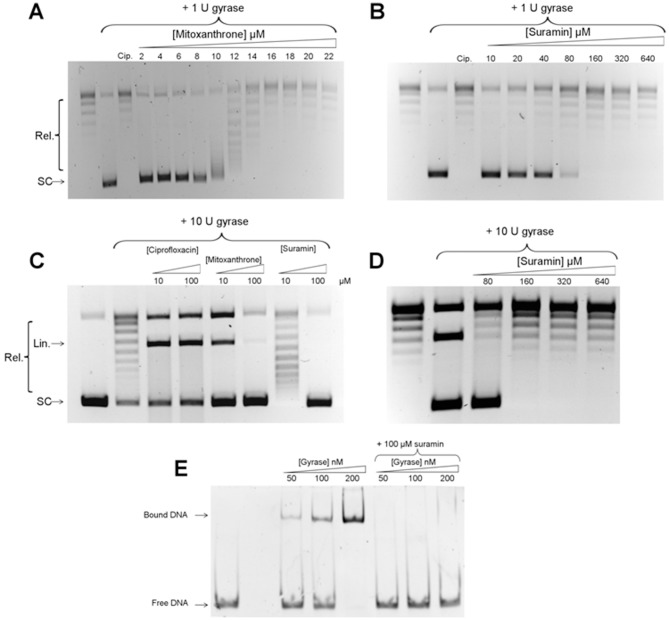
*In vitro* characterisation of DNA gyrase screen hits. A. Determination of the IC_50_ for mitoxantrone in a supercoiling assay with 1 unit of gyrase (12 nM); 100 μM ciprofloxacin (Cip.) was used as a positive control for inhibition. The positions of relaxed (Rel.) and negatively supercoiled (SC) DNA are indicated. B. Determination of the IC_50_ for suramin. C. Assaying the abilities of mitoxantrone and suramin to induce gyrase-mediated DNA cleavage. The reactions were carried out in the absence of ATP. Ciprofloxacin was used as a positive control for gyrase-mediated cleavage. The position of linear DNA (Lin.) is indicated. D. Suramin-induced protection of DNA from Ca^2+^-induced, gyrase-mediated cleavage. E. Inhibition of gyrase binding to a 147 bp DNA fragment by suramin.

Out of the 13 hits identified in the gyrase screen, two were novel gyrase inhibitors: mitoxantrone and suramin. Both of these compounds have previously been shown to have activity against eukaryotic topo II [55], [58], but they had not previously been shown to be active against DNA gyrase. The IC_50_ values for these compounds against *E. coli* DNA gyrase were determined to be 12 µM for mitoxantrone and 80 µM for suramin in the gel assay (Figures 3A and 3B).

Mitoxantrone is from the anthraquinone class of drugs and is currently used as an antineoplastic agent [59]. It is thought to inhibit topo II by stabilisation of the DNA-cleavage intermediate, leading to generation of double-stranded breaks in DNA [58]. To determine if this mode of action is the same for its inhibition of gyrase, a gel-based DNA cleavage assay was conducted under conditions which reveal formation of the cleavage intermediate (Figure 3C). It was observed that mitoxantrone strongly induced DNA cleavage by DNA gyrase at 10 µM, comparable to the known cleavage-intermediate stabiliser ciprofloxacin, showing that mitoxantrone stabilises the cleavage complex of gyrase as well as topo II. This is likely to be due to the drug intercalating at or near the DNA break sites generated in the cleavage complex in both enzymes. It also appears that at 100 µM the drug's ability to stabilise the cleavage complex is reduced; this is probably due to its binding to DNA and inhibiting enzyme binding. Suramin, on the other hand did not display any ability to induce cleavage.

Suramin is an anti-protozoal drug that has been subjected to clinical trials for the treatment of several forms of cancer [60]. Although it has been shown to protect against cleavage of DNA by topo II induced by cleavage-intermediate stabilising agents [55], its exact mode of inhibition has yet to be determined [61]. The ability of suramin to protect DNA from gyrase-induced cleavage was tested (Figure 3D). To eliminate the possibility of drug-drug interactions, Ca^2+^ was used to induce cleavage by DNA gyrase [62]. Suramin at 80 µM was able to completely protect DNA from cleavage by gyrase in the presence of 4 mM calcium chloride, indicating that its mode of action is similar to that found with topo II and is independent of drug-drug interactions.

To determine if the drug was protecting from cleavage by preventing binding of the protein to DNA, a native gel-shift assay to measure the binding of DNA gyrase to a 147 bp DNA fragment in the presence or absence of 100 µM suramin was carried out (Figure 3E). In the absence of suramin, the conversion of free DNA to a slower-migrating form was observed as enzyme was titrated in, whereas the presence of 100 µM suramin abolishes this shift. This suggests that the drug is preventing the binding of the enzyme to DNA rather than preventing cleavage directly, similar to the antibiotic simocyclinone D8 [33].

**Figure 4:**
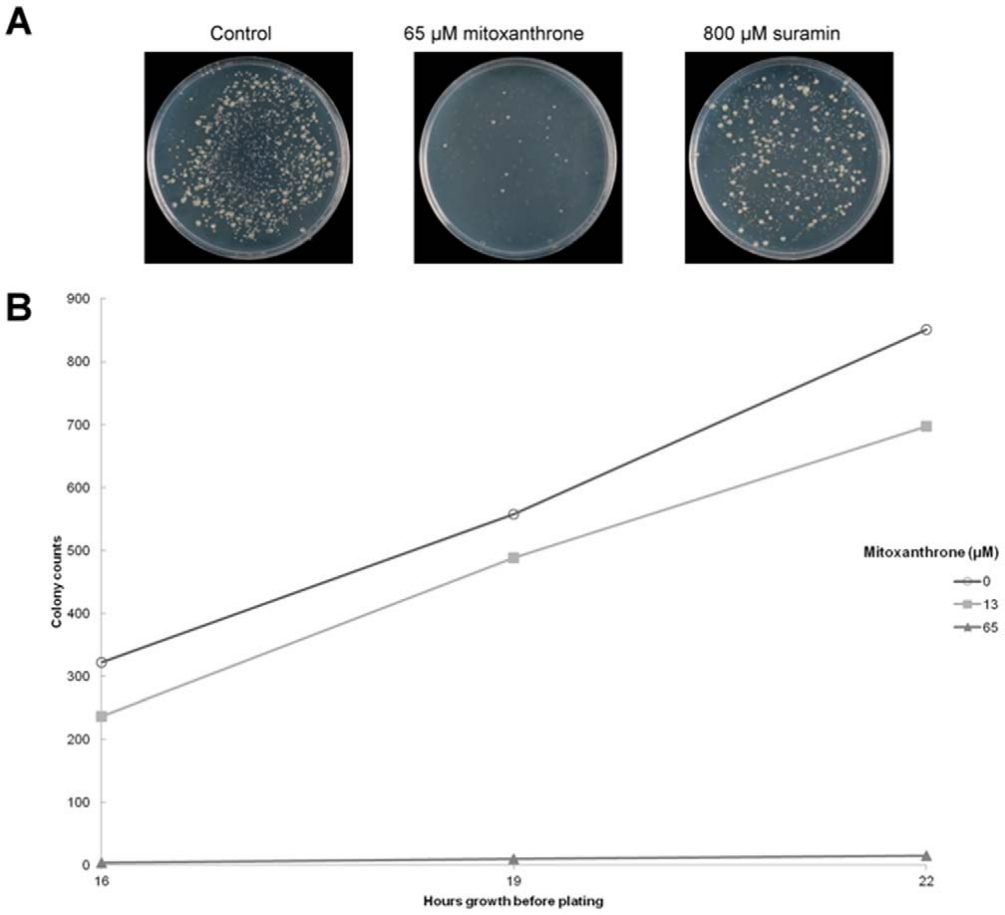
Growth of *M. smegmatis* mc^2^155 in the presence of mitoxantrone or suramin. A. Bacteria were grown in liquid cultures in the presence of drug for 22 hours, and then plated onto drug-free agar. After a further 48 h the colonies were counted for each concentration of drug. B. Colony counts for bacteria grown in the presence of 0, 13 or 65 µM mitoxantrone for 16, 19 or 22 hours before being plated onto solid media.

The antimicrobial activities of these two compounds were tested against both Gram-negative (*E. coli* MG1655 [63]) and Gram-positive bacteria (*M. smegmatis* mc^2^155). Additionally the growth of the membrane-permeable *E. coli* strain NR698 in the presence of the drugs was investigated [64]. (NR698 carries an in-frame deletion for the *imp* gene (*imp4213*), the product of which is responsible for lipopolysaccharide assembly in the outer membrane [65]. This mutation causes the outer membrane of the bacteria to become leaky, making it more sensitive to antimicrobials.) Although neither drug displayed any inhibition of growth on any of the bacterial strains tested when they were grown on solid media, mitoxantrone strongly inhibited the growth of *M. smegmatis* in liquid broth. Bacteria were initially grown in liquid media in the presence or absence of drug before being plated onto drug-free agar plates. The number of colonies produced was counted and used as an indication of drug efficiency. For 65 μM mitoxantrone the colony count was only 2% of what was recorded in the absence of drug (Figure 4).

### 9-Aminoacidine, hexylresorcinol, mitoxantrone, purpurin, quinacrine and suramin are novel inhibitors of *M. mazei* and *S. shibatae* topoisomerase VI that prevent DNA cleavage

**Figure 5:**
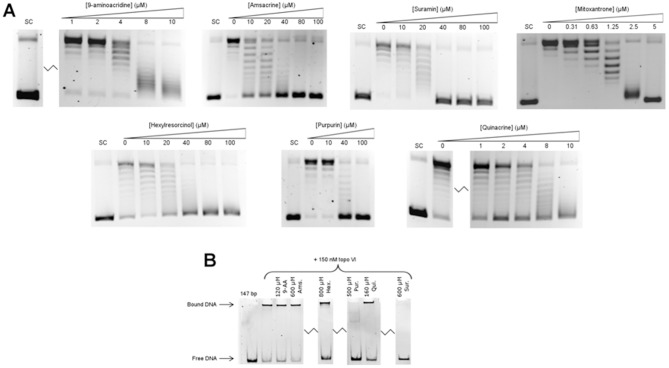
*In vitro* characterisation of topoisomerase VI screen hits. A. Determination of IC_50_ values for hits in the presence of 1 unit (50 nM) of *M. mazei* topo VI. This resulted in the following IC_50_ values: 6 μM for 9-aminoacridne; 30 μM for m-amsacrine; 30 μM for suramin; 30 μM for hexylresorcinol; 40 μM for purpurin; 8 μM for quinacrine; and 2 μM for mitoxanthrone. B. Native gel shift assays for the binding of *M. mazei* topo VI to a 147 bp DNA fragment in the presence of screen hits.

Having identified 6 novel inhibitors of *M. mazei* topo VI, their mechanism of action was investigated using the gel-based relaxation assay. The IC_50_ values of the compounds were determined by titrating the various compounds into reactions containing 1 unit of topo VI (Figure 5A). The most potent hit was mitoxantrone, with an IC_50_ of 2 µM, while the least potent inhibitor was hexylresorcinol, with an IC_50_ of 40 µM. The IC_50_ of suramin was estimated to be 30 µM with *M. mazei* topo VI whereas it had previously been shown to have an IC_50_ of 80 µM with *E. coli* DNA gyrase.

To test if any of the hits inhibited *M. mazei* topo VI by preventing the binding of the enzyme to DNA, native gel shift assays were carried out with *M. mazei* topo VI in the presence of the screen hits (Figure 5B). Out of the 6 compounds both suramin and purpurin appeared to prevent the binding of topo VI to DNA. Since it had already been determined that suramin had a similar mechanism of action with *E. coli* DNA gyrase it was likely that this result was genuine, and suramin was excluded from further mechanistic tests. For the sample containing purpurin, two faint bands part way into the gel were observed. This could indicate that the drug is actually causing the subunits of the enzyme to dissociate rather than preventing the binding of DNA directly. As such, these bands could signify DNA bound to topo VIA subunit monomers or dimers.

All hits were tested in ATPase assays (data not shown). Controls were included in which the enzyme and hit were incubated in the absence of ATP to account for any non-specific effects of the drug. None of the hits displayed significant inhibition of *M. mazei* topo VI ATPase activity, although hexylresorcinol appears to stimulate hydrolysis of ATP by topo VI; his may indicate drug-induced uncoupling of ATP hydrolysis and relaxation activity.

**Figure 6:**
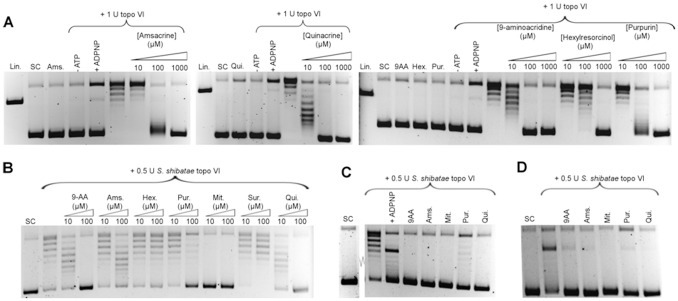
DNA cleavage assays with topoisomerase VI screen hits. A. Assaying the abilities of screen hits to induce *M. mazei* topo VI-mediated DNA cleavage with 1 unit topo VI (50 nM). B. Inhibition of *S. shibatae* topo VI by screen hits. C. Assaying the abilities of screen hits to induce *S. shibatae* topo VI-mediated DNA cleavage. D. Protection of DNA from ADPNP-induced, *S. shibatae* topo VI-mediated cleavage by screen hits.

The ability of the hits to stabilise the cleavage complex of *M. mazei* topo VI was tested as described for the DNA gyrase screen hits. In contrast to *S. shibatae* topo VI [53], *M. mazei* topo VI appeared to be resistant to cleavage induced by both the non-hydrolysable ATP analogue ADPNP and CaCl_2_ (Figure 6A and data not shown). Denaturing with increased amounts of SDS (2%), 170 µM NaOH (pH 11 final), 600 µM guanidinium hydrochloride or 800 µM urea did not improve the amount of cleavage, nor did increasing the proteinase K digestion time (data not shown). *M. mazei* topo VI was shown by SDS-PAGE to have been fully digested by proteinase K under the standard cleavage conditions (data not shown).

The abilities of the screen hits to stabilise the cleavage complex were tested by using the standard conditions for the cleavage assay (Figure 6A). To test if the compounds had any intrinsic DNA cleavage activity, control reactions were included containing plasmid pNO1 and the highest concentration of each drug tested with no enzyme. To establish the level of background cleavage, a control was included containing topo VI in the standard reaction buffer but lacking ATP. As a positive control for cleavage, a reaction was included containing topo VI and 1 mM ADPNP. No increase was observed in DNA cleavage above the background with any of the compounds, which suggests that none of the compounds tested stabilise the cleavage complex. However, the level of cleavage observed in the positive control containing ADPNP was very low when compared to similar experiments with *E. coli* DNA gyrase (Figure 3C).

Since it had proved difficult to achieve clear results for cleavage-complex stabilisation of *M. mazei* topo VI by the screen hits, we also tested them against the *Sulfolobus shibatae* enzyme. Cleavage complex stabilisation with *S. shibatae* topo VI by ADPNP and CaCl_2_ has been demonstrated previously [53]. Out of the *M. mazei* topo VI screen hits, hexylresorcinol and suramin did not display any inhibition of *S. shibatae* topo VI (Figure 6B). The compounds that were inhibitors of *S. shibatae* topo VI were then tested in the cleavage assay at concentrations predicted to give complete inhibition (Figure 6C). ADPNP was used as a positive control for cleavage-complex stabilisation and showed a good level of cleavage. However no induction of cleavage by any of the screen hits was observed.

To test if any of the inhibitors operated by preventing the cleavage of DNA by *S. shibatae* topo VI, cleavage assays were carried out in the presence of ADPNP with or without the compounds (Figure 6D). A reduction in intensity of the linear band would indicate that the drug is able to prevent ADPNP-induced cleavage of the DNA by topo VI. All five compounds displayed a marked ability to reduce the amount of cleavage, suggesting either they were interfering with the binding of ADPNP or DNA, or inhibiting the cleavage reaction. This was expected for purpurin, which was shown in earlier experiments to prevent the binding of *M. mazei* topo VI to DNA but was surprising for the other compounds, none of which had shown any ability to affect DNA binding. It may be that these compounds are capable of inhibiting the cleavage reaction itself.

### Inhibition of *A. thaliana* growth by hexylresorcinol is consistent with inhibition of topoisomerase VI *in planta*

To date, *Arabidopsis* topo VI has not been expressed in a soluble form suitable for enzymology experiments. However, knock-out mutants of topo VI in *Arabidopsis* have been shown to have a very clear “dwarf” phenotype [39], [40], [66]. This arises from the fact topo VI is thought to be involved in the process of endoreduplication in plants, which is in turn linked to cell expansion [67]. Plants lacking topo VI are generally smaller than wild type and have reduced cell size and ploidy (chromosome count). It is therefore possible to assay for compounds that inhibit *Arabidopsis* topo VI *in vivo* by looking for these characteristics.

The ability of the hits from the *M. mazei* topo VI screen to inhibit the growth of *Arabidopsis* seedlings in a hypocotyl extension assay [40] was tested. The length of *Arabidopsis* seedlings grown in the presence of the screen hits was measured using a light microscope and compared to control plants grown in the absence of drug. Out of the hits tested, only hexylresorcinol displayed any effect on plant growth, completely preventing seed germination at 100 µM.

**Figure 7:**
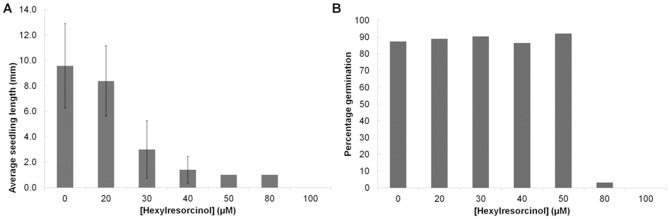
Inhibition of *Arabidopsis* hypocotyl extension by hexylresorcinol. All plants were grown for 5 days in the dark. A. Average length of seedlings grown on 20, 30, 40 50, 80 or 100 µM hexylresorcinol. Error bars represent the standard deviation of the samples. B. Percentage germination of seedlings grown on 20, 30, 40 50, 80 or 100 µM hexylresorcinol.

To explore this further, the ability of hexylresorcinol to inhibit plant growth was tested at a range of concentrations. For each concentration the average hypocotyl length and the percentage of seeds that had germinated was calculated. It was observed that the number of seeds germinated remained constant up to 50 µM hexylresorcinol, while the average length of the seedlings dropped rapidly (from an average of 10 mm in the absence of drug to approximately 1 mm with 50 µM hexylresorcinol). At 80 µM germination was reduced, whilst no seeds germinated at 100 µM (Figure 7).

**Figure 8:**
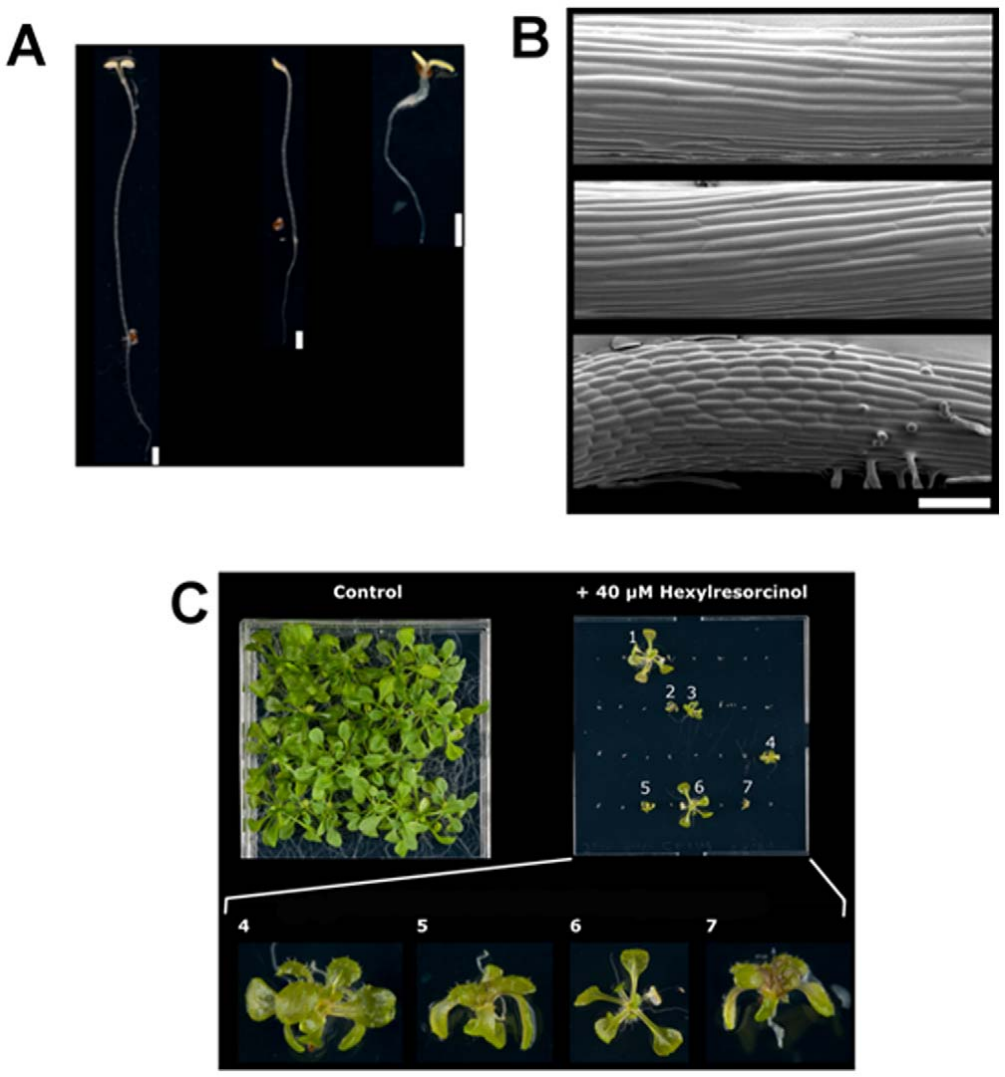
Activity of hexylresorcinol on *Arabidopsis thaliana* col-0. A. Hypocotyl extension assay (1 mm scale bars). Left: control plant grown in the absence of hexylresorcinol. Centre: plant grown on 40 μM hexylresorcinol exhibiting normal morphology. Right: plant grown on 40 μM hexylresorcinol exhibiting dwarf morphology. B. Cyro-electron microscopy images of hypocotyls (100 μm white scale bar). Top: control plant grown in absence of hexylresorcinol. Middle: plant grown on 40 μM hexylresorcinol exhibiting normal morphology. Bottom: plant grown on 40 μM hexylresorcinol exhibiting dwarf morphology. C. Three-week-old *Arabidopsis* plants grown on 40 μM hexylresorcinol. A control plate which did not contain the compound was also included. Photos at a higher magnification were taken of plants displaying different morphologies. Plants 2, 5 and 7 displayed the “dwarf” morphology, whilst plants 1 and 6 were similar to the control in appearance. Plants 3 and 4 appeared intermediate in morphology. All hexylresorcinol-grown plants appeared somewhat transparent compared to the control plants.

Seedlings germinated at 50 µM were too small to discern any differences in morphology, so further studies involved plants grown at 40 µM. At that concentration we observed a range of responses to the compound (Figure 8A). Although the average hypocotyl length was considerably reduced, a few plants appeared to be unaffected by the drug, reaching similar hypocotyl lengths as plants grown in the absence of compound. Out of the shorter plants some appeared to have normal morphology (apart from their reduced size) whilst others were very short with fatter hypocotyls and reduced root hair length, the latter matched the description of topo VI knock-out mutants. These plants were therefore designated as having “dwarf” morphology.

To further explore the morphological effects of the drug, plants grown on 40 µM hexylresorcinol were allowed to mature into a rosette (Figure 8C). Although the majority of the seeds germinated, only seven of the 32 plants were able to develop into a rosette. These plants appeared pale, yellowish and slightly transparent compared to control plants grown without drug. Some of the plants (plants 1 and 6) displayed normal morphology and were close to the control in size. One plant (plant 3) displayed normal morphology, but was greatly reduced in size suggesting its growth was slowed. The remaining plants (plants 2, 4, 5 and 7), which had displayed the “dwarf” morphology of hypocotyls, were very small with shortened leaf stems. However, in contrast to published reports of topo VI knock-out plants [40], they appeared to have normal-sized trichomes. Plants exhibiting normal morphology recovered fully when transferred to fresh agar plates, although some leaves appeared white and shrivelled. In contrast plants that displayed “dwarf” morphology experienced limited recovery, remaining about half the size of -plants with normal morphology and did not display any withering.

To see if the reduction of size in the “dwarf” plants was due to a reduction in cell size, rather than a reduction in the number of cells, Cryo-Scanning Electron Microscopy (Cryo-SEM) was conducted on *Arabidopsis* hypocotyls grown for 5 days in the dark. It was observed that the hypocotyls of control seedlings grown without hexylresorcinol were comprised of cells ∼300 µm in length (Figure 8B). Seedlings that had normal morphology when grown on 40 µM hexylresorcinol had cell lengths very similar to the control. In contrast, seedlings which displayed the “dwarf” morphology had drastically reduced cell sizes of ∼50 µm. These results suggested that the reduced size of the “dwarf” plants was due to a reduction in cell size rather than number, an observation consistent with the hypothesis that hexylresorcinol targets topo VI in the plants.

## Discussion

DNA topoisomerases are important drug targets, being both essential and ubiquitous. Furthermore convergent and divergent evolution has resulted in diversity of topoisomerases amongst the various domains and kingdoms of life, enabling the selective inhibition by drugs of certain enzymes but not others. This approach has been used to great effect to produce antibacterials that target the bacterial enzyme DNA gyrase. Topo VI is an unusual enzyme, being the only known member of the type IIB family of topoisomerases, found in plants and archaea as well as being hypothetically present in plasmodia [39], [42], [68]. As such, specific inhibitors of this enzyme could be used as novel herbicides and potential antimalarials, as well as provide insight into the enzyme's mechanisms of action. With the emergence of strains of various pathogens resistant to many clinical antibiotics, there is a pressing need to discover compounds that inhibit topoisomerases in novel ways. However, high-throughput screening for these compounds has been limited by the traditional assays for topoisomerase activity which are poorly suited to rapidly processing large numbers of reactions in a quantitative manner.

In previous work we have shown that it is possible to assay DNA topoisomerases using a microtitre plate-based assay that is based on the preferential formation of DNA triplexes in supercoiled over relaxed DNAs [46]. In this work we have successfully validated the assay for screening compound libraries against DNA topoisomerases. Although the screens in this study were conducted with a relatively small number of compounds, the 96-well plate format and quantitative output of topoisomerase activity allows the possibility of automation and thus the capacity to screen larger libraries. For comparison, to reproduce the screens presented in this work using the traditional gel-based assay for topoisomerases would require 72×64-well agarose gels, which would have to be manually stained, destained and inspected. The overall quality of the data for the screen was good, and the assay was able to identify both known and novel inhibitors using these screens.

Cross-checking the hits from the DNA gyrase and topo VI screens revealed that mitoxantrone, a hit in the DNA gyrase screen and a DNA intercalator, was also an inhibitor of *M. mazei* topo VI, which had been missed in the topo VI screen. It was likely that mitoxantrone had been overlooked because of its ability to disrupt triplex formation, which results in a decrease of fluorescence signal in the assay which can be misinterpreted as relaxation activity (data not shown). Indeed drugs that disrupt triplex formation could lead to false positives in the gyrase assay. This illustrates a potential weakness of the assay: if a compound inhibits or promotes triplex formation this may mask their ability to inhibit topoisomerases; this will particularly be an issue with intercalators, which are quite often topoisomerase inhibitors.

Out of the two novel hits from the DNA gyrase screen, suramin displayed no antimicrobial activity with any of the bacterial strains tested, whereas mitoxantrone was found to inhibit the growth of *M. smegmatis* in liquid cultures at 65 µM. However, no inhibition of growth was seen with either wild-type *E. coli* or the more permeable strain NR698. This was true for bacteria grown on both liquid and solid media, suggesting that either the drugs cannot gain entry to the bacteria, despite the mutation present in NR698, or that *E. coli* is naturally resistant to them (e.g. via an efflux pump, a modification or degradation pathway).

The reason why mitoxantrone can inhibit bacterial growth only in liquid cultures may be because the bacteria exist in a very different growth state while they are growing on solid media. It has been shown that bacteria growing in biofilms can be more resistant to antimicrobial agents than free-floating planktonic cells [69]. Reasons why this might be are numerous, including: the colony preventing diffusion of drug, slower growth rate of the bacteria on the solid media and the induction of biofilm-specific stress responses and phenotypes.

Interestingly mitoxantrone and suramin were inhibitors of both *E. coli* DNA gyrase and *M. mazei* topo VI. Both of these compounds have also been reported as inhibitors of eukaryotic topo II [55], [58], a type IIA topoisomerase. It has been previously described that topo VI appears to be more susceptible to topo II inhibitors than it is to specific inhibitors of DNA gyrase [44]. This is true for the majority of topo VI hits described in this work with m-amsacrine, quinacrine and 9-aminoacridine all being implicated in topo II inhibition [70], [71]. What is interesting about mitoxantrone and suramin is that they appear to inhibit DNA gyrase, topo II and topo VI (although suramin did not inhibit *S. shibatae* topo VI). This broad-ranging inhibition probably means they target fundamental aspects of the type II topoisomerase reaction. In the case of suramin this appears to relate to the binding of the enzymes to DNA, possibly by acting as a mimic of the phosphate-based backbone of DNA via its sulfonic acid groups. For mitoxantrone this is likely to be due to intercalation at or near the double-strand break in the G-segment DNA, since it is able to stabilise the cleavage complex of both gyrase and eukaryotic topo II [58]. It was therefore surprising that DNA cleavage was not detectable with either *M. mazei* or *S. shibatae* topo VI. In the case of *M. mazei* topo VI this may be explained by the difficulty in revealing the cleavage complex with this enzyme, but this does not hold true for the *S. shibatae* enzyme for which cleavage with ADPNP was observed.

For the majority of the *M. mazei* topo VI inhibitors, except for suramin and purpurin, which both appeared to prevent G-segment binding, it was not possible to conclusively determine mechanisms of action. They did not appear to inhibit the ATPase activity of the enzyme, prevent G-segment binding or stabilise the cleavage complex with *M. mazei* topo VI. Out of those that inhibited *S. shibatae* topo VI none appeared to stabilise the cleavage complex. However mitoxantrone, quinacrine and 9-aminoacridine all appeared to prevent ADPNP-induced cleavage of DNA by the enzyme; both quinacrine and 9-aminoacridine have been reported to prevent the cleavage of DNA by eukaryotic topo II in fibroblasts [72]. It could be that the mechanism of action for these compounds against topo VI is their ability to prevent the formation of the cleavage complex by interacting in the DNA-protein complex in such a way to distort the DNA and make it unsuitable for cleavage. Hexylresorcinol appeared to increase the rate of ATP hydrolysis by *M. mazei* topo VI, which may be indicative of the compound uncoupling ATP hydrolysis and DNA cleavage.

Several of the compounds identified by the screen are members of the acridine class of compounds, which have been shown to have efficiency against *P. falciparum*, suggesting that these drugs may also target the *Plasmodium* orthologue of topo VI. Quinacrine is a well-established anti-malarial drug, but its mode of action remains unclear. It has been shown to target the erythrocyte stage of the *Plasmodium* life cycle, during which the parasite undergoes a process similar to endoreduplication called schizogony [56], [73], [74]. Derivatives of amsacrine have been also shown to have potency against *Plasmodium falciparum* [75]. These links raise the possibility that topo VI is a target for these drugs.

Out of the hits from the *M. mazei* topo VI screen, hexylresorcinol was shown to have a significant effect on the growth of *Arabidopsis thaliana*. (No other compounds inhibited plant growth; it is not clear why this is so but it could be due to differences in *A. thaliana* and *M. maze*i topo VI enzymes or poor uptake of the drugs by the plant.) Treating plants with 40 µM hexylresorcinol resulted in an overall decrease in plant size and caused plants to become pale and transparent. At this concentration there appeared to be a range of responses to the drug with some plants largely unaffected, some with slowed growth and others exhibiting a reduction in overall size and root hair size and frequency (the “dwarf” morphology). The characteristics of the “dwarf” morphology are similar to those observed in topo VI knock-out mutants [39], [40]. Additionally all plants took on a yellowish colour, which was noted in some of the topo VI mutants [39]. However, it appears that the size of the trichromes remains largely unaffected, whereas they were reduced in the topo VI mutants.

Plants exhibiting the “dwarf” phenotype failed to recover fully when transferred to fresh agar lacking hexylresorcinol. After three weeks the plants that had either not been affected or had their growth slowed had mostly recovered, growing to full size and regaining their pigmentation. Interestingly, a few leaves on these plants turned white and withered. In contrast, plants that had exhibited the dwarf morphology remained smaller and did not have any withered leaves, although they did grow considerably and retain their pigmentation. These observations suggest that the withering of leaves is an immune response to the drug, most likely sequestering the herbicide into certain leaves. A similar response has been reported for weeds resistant to the common herbicide glyphosate [76]. It is possible that plants which are able to sequester hexylresorcinol successfully are able to grow normally or have their growth slowed as a stress response when grown on sub-lethal concentrations of drug, whereas those that cannot do so have inhibited topo VI activity and therefore exhibit the “dwarf” morphology.

To determine whether the “dwarf” morphology was due to *in vivo* inhibition of endoreduplication, cryo-SEM was carried out on seedlings grown in the presence of hexylresorcinol. Since endoreduplication results in increased cell expansion the expected result for its inhibition is a reduction in cell size. Plants that had grown normally or slowly both exhibited a cell size of around 300 µm whereas plants exhibiting the “dwarf” phenotype were reduced to around 50 µm. As to whether this decrease in endoreduplication is due to the inhibition of *Arabidopsis* topo VI *in vivo,* it is difficult to say for certain since there is always the possibility that hexylresorcinol affects other enzymes involved in the process. The drug does provoke other morphologies (such as reduction in pigmentation) that are not solely explainable as an effect of reduced endoreduplication. It is therefore possible that it has multiple targets within the plant. Additionally, although the “dwarf” morphology shares some of the features with the topo VI knock-out mutants (overall size reduction, yellowish colour, and reduced root hair size and frequency), there are some key differences. Trichome size appears to be unaffected, whereas it was reduced in the mutants, which suggests that hexylresorcinol is targeted to specific tissues within the plant. Taken together the results show that hexylresorcinol leads to a dwarf morphology that is consistent with an effect on endoreduplication via topo VI inhibition, but further work would be required to establish this.

One way to prove that topo VI is the target within the plant would be to express and purify the plant enzyme and demonstrate that hexylresorcinol inhibits it *in vitro*; our efforts to purify active plant topo VI have so far been unsuccessful (data not shown). Alternatively plants resistant to hexylresorcinol could be selected and the resistance genes mapped and sequenced. Our observations that the drug inhibits *M. mazei* topo VI *in vitro* and *Arabidopsis* endoreduplication *in planta,* provides at least circumstantial evidence for topo VI being the *in vivo* target for this compound. Hexylresorcinol is an active ingredient of acrisorcin (Akrinol), a topical antifungal [77] and has been used as an oral antiseptic [78], and is an active ingredient in throat lozenges, but there has been no previous reports of its herbicidal activity. Hence, the significance of its effect on *Arabidopsis* and on topo VI remains to be established.

The emergence of pathogenic bacterial strains resistant to currently used antibiotics is a problem of growing importance. Topoisomerases have proved to be extremely successful targets for anticancer and antibacterial drugs, but the search for novel inhibitors of these enzymes has been hampered by the low-throughput nature of traditional assays. In this work we have validated a novel assay for topoisomerase activity by successfully screening a small library of compounds against *E. coli* DNA gyrase and *M. mazei* topo VI. Several novel inhibitors for these enzymes have been identified and their mechanisms of action explored *in vitro* and *in vivo*. This work provides a blueprint for larger-scale screens for inhibitors of topoisomerases which have the potential to lead to new therapeutics.

## Materials and Methods

### Enzymes, DNA and compounds

*E. coli* DNA gyrase and his-tagged *M. mazei* topo VI were prepared as described previously [36], [79]. *S. shibatae* topo VI was a gift from Danielle Gadelle (University of Paris XI, Orsay). One unit of enzyme is defined as the amount required to fully supercoil (in the case of *E. coli* DNA gyrase) or relax (in the case of *M. mazei* topo VI) 0.5 µg of pNO1 in 30 min. at 37°C. In the case of *S. shibatae topo* VI, one unit was defined as the amount required to fully relax 0.5 µg of pNO1 in 5 min at 75°C. Wheat germ topo I was purchased from Inspiralis Ltd (Norwich, UK). Chicken erythrocyte topo I was a gift from Alison Howells (Inspiralis Ltd.).

Plasmid pNO1 is a modified version of plasmid pBR322* containing a 16 bp triplex forming sequence (5′-CTCTCTCTCTCTCTCT) [46]. Supercoiled plasmid was purified by caesium chloride gradient [80]. Relaxed substrate was prepared by incubating 10 mg of supercoiled plasmid with chicken erythrocyte topo I for 1 h at 37°C in 50 mL of: 20 mM Tris·HCl (pH 8.0), 200 mM NaCl, 0.25 mM EDTA, 5% glycerol. The relaxed plasmid was subsequently purified by phenol-chloroform extraction and ethanol precipitation. The biotinylated oligonucleotide TFO1 (5′ biotin-TCTCTCTCTCTCTCTC) was synthesised by Sigma-Aldrich Co. LLC.

Screening was carried out on the GenPlus library from Microsource Ltd. Daughter plates were prepared with a compound concentration of 250 µM in 100% DMSO. Fresh stocks of potential hits were ordered either from Sigma-Aldrich Co. LLC. or Microsource Ltd.

### DNA triplex-based assay for topoisomerase activity

The triplex-based assay was conducted as previously described [45]–[47]. In brief, oligonucleotide TFO1 was immobilised onto a streptavidin-coated microtitre plate. Excess oligonucleotide was removed by washing with buffer. Topoisomerase reactions were carried out in a 30 μL reaction volume containing 1–2 units topoisomerase and 1 μg relaxed or negatively supercoiled pNO1 under the published reaction conditions [36], [47]; gyrase supercoiling: 35 mM Tris•HCl (pH 7.5), 24 mM KCl, 4 mM MgCl_2_, 2 mM DTT, 1.8 mM spermidine, 1 mM ATP, 6.5% (w/v) glycerol, 0.1 mg/mL bovine serum albumin; topo VI relaxation: 20 mM bis-tris propane (pH 6.5), 100 mM potassium glutamate, 10 mM MgCl_2_, 1 mM DTT, 1 mM ATP. The reaction was stopped by the addition of a low pH, high salt buffer and triplex formation was allowed to occur for 30 min at room temperature. The DNA retained on the plate was stained with SYBR Gold (Sigma) dye and the fluorescence readings for each well read in a SpectraMax Gemini fluorimeter (Molecular Devices).

### Topoisomerase screening conditions

Twelve plates were screened in duplicate over three days manually using a multichannel pipette as previously described [45]. Each reaction contained a final concentration of approximately 25 µM compound and 5% DMSO. Each plate contained 16 control reactions: eight positive for enzyme activity, which contained enzyme but no library compounds, and eight negative for enzyme activity, which lacked both enzyme and library compounds. All controls were carried out with 5% DMSO. Once the data had been collected from the screen, the fluorescence signals for the duplicates were averaged and converted into percentage inhibition using the positive and negative controls. An arbitrary hit threshold of 25% inhibition was used to select compounds for validation by the agarose gel-based assay. To analyse the degree of separation between a negative and positive result the Z' factor [51] for the assay was calculated from the data for the control reactions (Equation 1).
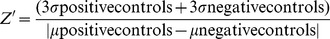
(1)


### Agarose gel-based assay for DNA topoisomerase activity

The agarose gel-based assay was conducted as previously described [36], [44], [62]. In brief, topoisomerases were incubated with 0.5 µg of plasmid and the desired concentration of compound for 30 min at 37°C (for DNA gyrase and *M. mazei* topo VI) or 5 min at 75°C (for *S. shibatae* topo VI), in a total volume of 30 µL. The final concentration of DMSO in these reactions did not exceed 5%. Reactions were stopped by the addition of a loading buffer containing EDTA and the drug extracted by vortexing with either chloroform or aqueous butanol as required. In the case of *M. mazei* topo VI, it was necessary to include SDS in the loading buffer, at a final concentration of 1%. Samples were loaded onto a 1% agarose gel, run for the appropriate length of time and stained with ethidium bromide before visualisation on a UV transilluminator.

### Agarose gel-based assay for DNA cleavage by DNA topoisomerases

Assays to detect the stabilisation of cleavage complexes were identical to the protocol for topoisomerase activity detailed above, except as follows. Cleavage experiments with DNA gyrase were carried out in the absence of ATP and spermidine and 30 µL reactions were stopped with 3 µL 10% SDS and incubated with 3 µL 1 mg/mL proteinase K solution at 37°C for 1 h. For cleavage complex protection assays with suramin, the reaction buffer was modified to contain 4 mM CaCl_2_ rather than MgCl_2_.

### Pyruvate kinase-linked assay for topoisomerase ATPase activity

ATPase assays were performed essentially as described previously [81] except they were adapted to a microplate format using clear, colourless 96-well Microtitre plates (Pro-bind™, Becton Dickinson). Reactions were carried out in 100 µL of the established reaction buffer for either DNA gyrase of *M. mazei* topo VI [36], [62] supplemented with: 800 µM phosphoenolpyruvate (PEP), 400 µM NADH, 1% (vol/vol) PK/LDH (pyruvate kinase-lactate dehydrogenase mixture in 50% (w/v) glycerol,100 mM KCl, 10 mM HEPES (pH 7.0)) and the desired concentration of hit compound. ATP was initially withheld from the reaction. After 5 min incubation at room temperature the reactions were initiated by the addition of ATP and the absorbance at 340 nm was measured over the course of an hour using a Spectra Max Plus absorbance reader (Molecular Devices). Data were processed by omitting the first 10–15 min of collection and normalising the first retained time point. This was done to exclude artifacts observed within this time frame and for ease of comparison of rates. Controls lacking topoisomerase were used to assess the intrinsic ATPase activity of the test compounds.

### Electrophoretic mobility shift assay for topoisomerase-DNA binding

In order to test the effects of hit compounds upon DNA binding by topoisomerases, samples of enzyme and drug were prepared under the following conditions in a final volume of 10 μL: 1 nM 147 bp linear fragment of DNA derived from pBR322, [82] 50 mM Tris•HCl (pH 7.5), 100 mM KCl, 5 mM MgCl_2_, 2 mM DTT, 10% w/v glycerol. Samples were incubated for 30 min at room temperature before being run on a 5% (29:1) Protogel acrylamide (National Diagnostics) gel in 90 mM Tris·Borate, 5 mM MgCl_2_ at 150 V for 45 min. The DNA was stained by soaking in 2 µg/mL ethidium bromide for 10 min and visualised under UV light.

### Assaying *E. coli* DNA gyrase hits for bactericidal activity

For *E. coli* work, 10 mL cultures of *E. coli* MG1655 or NR698 were grown overnight at 37°C in LB medium. A 100 µL sample was then added to 10 mL of LB medium containing the desired concentration of compound. The culture was then allowed to grow at 37°C for 5 h and its OD_600_ measured every hour. For *M. smegmatis*, a 50 mL culture was grown for 48 h in Middlebrooks 7H11 medium at 37°C. A 100 µL sample was then added to 10 mL of Middlebrooks 7H11 containing the desired concentration of compound. The culture was then allowed to grow at 37°C for 9 h and its OD_600_ measured every three hours. For colony counting experiments *M. smegmatis* was grown for 48 h in 50 mL of Middlebrooks 7H9 medium at 37°C. A 100 µL sample was then added to 10 mL of Middlebrooks 7H9 medium containing the desired concentration test compound and allowed to grow at 37°C overnight. After 16 h, 20 µL samples were taken from these cultures and diluted 1 in 10,000 with Middlebrooks 7H9 medium. Petri dishes containing Middlebrooks 7H11 agar were prepared and 100 µL of each diluted culture was spread out. The plates were subsequently stored at 4°C. This was repeated every three hours for six hours, after which the plates were transferred to 37°C for 48 h. The numbers of colonies for each time point were counted manually.

### *Arabidopsis* hypocotyl extension assay and plant growth conditions

The hypocotyl extension assay was conducted as previously described [40], [66]. MS Salts medium [83] (containing micro and macro elements including vitamins, pH 5.8) was supplemented with 1 g/L sucrose and 0.7 g/L phytagel (Sigma) before autoclaving. After the medium cooled to ∼50°C it was divided into 50 mL aliquots and compounds to be tested (or an equivalent amount of appropriate solvent for control plates) then added at the desired concentration under aseptic conditions. The media were then poured into separate 100 mm square Petri dishes and allowed to cool to room temperature in a laminar flow hood. *Arabidopsis thaliana columbia* (Col-0) seeds were surface sterilised with 5% bleach for 10 min immediately prior to use. The seeds were then washed three times with sterile water under a laminar flow hood. After surface sterilisation the seeds were planted in a grid pattern (32 seeds per dish) and the dishes sealed with surgical tape. The plates were transferred to 4°C and left for 64 h in the dark to vernalise, after which they were transferred to a 22°C growth cabinet. After 2 h of light exposure the plates were stacked vertically in the dark at 22°C to allow for hypocotyl extension along the agar surface. After 4–5 days the hypocotyls were observed using an optical microscope and their length measured. For experiments where the plants were allowed to form rosettes, the *Arabidopsis* seedlings were grown horizontally under a 16 h day in a 22°C growth cabinet for the indicated number of weeks.

### Cryo-scanning electron microscopy (SEM) on *Arabidopsis* hypocotyls

Five day old hypocotyls grown on media containing various concentrations of compound were frozen in nitrogen slush and loaded into a Philips XL30 SEM with a cryo stage installed. Samples were sputter coated with platinum and transferred into the microscope chamber for image collection.
